# Culture-Dependent and -Independent Methods Capture Different Microbial Community Fractions in Hydrocarbon-Contaminated Soils

**DOI:** 10.1371/journal.pone.0128272

**Published:** 2015-06-08

**Authors:** Franck O. P. Stefani, Terrence H. Bell, Charlotte Marchand, Ivan E. de la Providencia, Abdel El Yassimi, Marc St-Arnaud, Mohamed Hijri

**Affiliations:** Department of Biological Sciences, Centre sur la biodiversité, Université de Montréal, Montréal, Québec, Canada; North Carolina State University, UNITED STATES

## Abstract

Bioremediation is a cost-effective and sustainable approach for treating polluted soils, but our ability to improve on current bioremediation strategies depends on our ability to isolate microorganisms from these soils. Although culturing is widely used in bioremediation research and applications, it is unknown whether the composition of cultured isolates closely mirrors the indigenous microbial community from contaminated soils. To assess this, we paired culture-independent (454-pyrosequencing of total soil DNA) with culture-dependent (isolation using seven different growth media) techniques to analyse the bacterial and fungal communities from hydrocarbon-contaminated soils. Although bacterial and fungal rarefaction curves were saturated for both methods, only 2.4% and 8.2% of the bacterial and fungal OTUs, respectively, were shared between datasets. Isolated taxa increased the total recovered species richness by only 2% for bacteria and 5% for fungi. Interestingly, none of the bacteria that we isolated were representative of the major bacterial OTUs recovered by 454-pyrosequencing. Isolation of fungi was moderately more effective at capturing the dominant OTUs observed by culture-independent analysis, as 3 of 31 cultured fungal strains ranked among the 20 most abundant fungal OTUs in the 454-pyrosequencing dataset. This study is one of the most comprehensive comparisons of microbial communities from hydrocarbon-contaminated soils using both isolation and high-throughput sequencing methods.

## Introduction

Over the past few decades, human activities related to petroleum consumption have led to massive releases of both aliphatic and aromatic hydrocarbons, making these compounds some of the most ubiquitous environmental pollutants on Earth [1–3]. Among these are the polycyclic aromatic hydrocarbons (PAHs), which are of particular concern, given their persistence in the environment (especially in soil) and the potent carcinogenic, mutagenic, and teratogenic effects that these compounds have on living organisms [4–6]. Many isolated strains of bacteria and fungi can degrade at least some components of hydrocarbon contaminants in culture [7–11], which makes these two major soil microbial groups promising reservoirs of hydrocarbon-degrading activity.

A number of studies have shown that bioremediation, the use of living organisms to decontaminate polluted sites, is likely a feasible solution for treating these contaminants [11–13]. One approach to enhancing hydrocarbon bioremediation is the stimulation of indigenous hydrocarbon degraders, by supplying limiting nutrients, oxygen, and/or improving the physicochemical conditions of the polluted soil [13–15]. Alternatively, cultured hydrocarbon degraders can be used to degrade contaminants *ex situ*, or spiked into contaminated soils *in situ*, a process known as bioaugmentation [16–18]. Regardless of the approach taken, effective bioremediation hinges on our ability to study microbes that are indigenous to polluted sites. Culture-independent and-dependent methods for microbial community analysis have both been used frequently to describe microorganisms from hydrocarbon-contaminated environments.

For culture-independent analysis, high-throughput sequencing techniques [19,20] have revolutionized our view of microbial ecology, revealing hyperdiverse communities that are extremely responsive to hydrocarbon contaminants across a variety of environments [21–25]. Although culture-dependent methods generally recover a small portion of the diversity from soil environments, they are still a critical component of bioremediation development and research [26–30]. In addition to the potential *in situ* and *ex situ* applications of cultured isolates, microbial isolation allows *in vitro* assessments of isolate physiology and hydrocarbon degradation pathways and performance, providing a basis for annotating extensive metagenomic datasets, and helping to identify genes and/or organisms that could be useful in land reclamation.

Hydrocarbon-contaminated soils may be more amenable to comprehensive culture-dependent sampling than other soil environments, since hydrocarbon contamination often leads to a decline in microbial diversity [22,24,31,32], meaning that a lower sampling effort may be required to isolate a representative proportion of the active community. Hydrocarbon contaminants may also suppress certain sensitive groups [33] and tend to select primarily for subgroups of the *Actinobacteria* and *Proteobacteria* in affected soils [22,23]. Although these phyla are extremely diverse, they are some of the best represented among cultured isolates [34,35]. However, although the gap between culture-independent and culture-dependent analyses of soil microbial communities is often mentioned, few studies [36–38] have directly compared these approaches, and none has specifically investigated the biases associated with culturing both bacteria and fungi in a bioremediation context, despite the critical role of culturing in this field.

In this study, we used both nutrient-rich and impoverished media, supplemented with various types and concentrations of petroleum hydrocarbons, in order to assess the effectiveness of culture-based methods at recovering indigenous microorganisms from hydrocarbon-contaminated soils. We paired 454-pyrosequencing of bacterial 16S rDNA and the fungal ITS region with extensive culturing of bacteria and fungi, using sediment samples harvested from a basin that is highly contaminated with hydrocarbons, at the site of a former petrochemical plant. Since different substrates select for different groups of bacteria [39] and fungi [40], bacterial and fungal strains were isolated using seven different culture media to enhance the number of potential isolates. Although there are many approaches for cultivating soil bacteria and fungi [41], we chose to use basic nutrient-rich and impoverished agar plates, since these are still the most widely applied culturing method in microbiology. While we expected much lower richness in the cultured dataset, it was interesting to observe that current culturing methods do not capture most of the dominant microorganisms found in hydrocarbon-contaminated soils through 454-pyrosequencing. This was more apparent among the bacterial isolates than the fungal isolates. Even more surprisingly, a number of the cultured microorganisms were not identified at all in the 454-pyrosequencing data.

## Materials and Methods

### Ethics statement

No specific permits were required for the described field study. The land on which we conducted the phytoremediation field is privately owned by ConocoPhillips. ConocoPhillips gave permission for the study to be conducted on their land. This field study did not involve endangered or protected species.

### Experimental design and sampling

Sampling occurred at the site of a former petrochemical plant at Varennes, on the south shore of the St-Lawrence River near Montreal, Quebec, Canada (45°41'56"N, 73°25'43"W). The sampling site is contaminated by a variety of industrial waste products related to petrochemical processing that have been released over the last forty years. The site was permanently closed in 2008, and since 2010, it has been used extensively to study the potential of willow cultivars for the phytoremediation of hydrocarbons (for details see www.genorem.ca and [23,42]). Five plots of 300 m^2^ each were set up within a contaminated area of approximately 2500 m^2^. Each plot was subdivided into 12 sub-plots of 25 m^2^ each. In June 2011, two soil samples were collected at depths of 25 cm and 50 cm in each of the 12 sub-plots within each plot. The 24 soil samples from each plot were then pooled to obtain representative composite soil samples. Soil samples were chilled at 4°C during transport from the field to the lab, and were stored at -20°C until isolation and DNA extraction. A portion of each composite soil sample was analyzed for F1-F4 hydrocarbons (sum of all aromatic and aliphatic hydrocarbon compounds with chain lengths of C10-C50) by Maxxam Analytics (Montreal, Quebec, Canada) on June 2011, according to the protocol set forth by The Canadian Council of Ministers of the Environment. Results from hydrocarbon analyses (S1 Table) revealed an increasing contamination gradient from plots 1 through 5, which led us to classify the plots into three discrete contaminant levels: slightly contaminated (plots SC1, SC2), contaminated (plot C3), and highly contaminated (plots HC4, HC5).

### Culture-dependent (CD) sample processing

For each composite soil sample, one gram of soil was suspended in 9 ml of sterile distilled water and vortexed thoroughly. From this stock solution, serial dilutions were performed to 10^−7^. Aliquots of 100 μl from dilutions of 10^−3^ and 10^−4^ for fungi, and of 10^−6^ and 10^−7^ for bacteria, were plated in duplicate on each of the seven culture media. Bacteria were isolated on tryptic soy agar (TSA, containing 30 g / L of tryptic soy broth (TSB)) and impoverished TSA plates (1 to 15 g /L of TSB) containing various concentrations of diesel engine oil or crude oil, or that had been coated with crude oil (Table 1). Fungal strains were isolated on potato dextrose agar (PDA, containing 24 g/L of potato dextrose broth (PDB)) and impoverished PDA plates (1 to 12 g/L of PDB) containing various concentrations of diesel engine oil or crude oil, or that had been coated with crude oil (Table 2). Petri dishes were inoculated, inverted, and incubated at 27°C for bacteria and 25°C for fungi. Bacterial colonies were checked every 48 hours for six days, and each new morphotype was subcultured on TSA for 48 hours. Fungal colonies were checked every 5 days for two weeks, and were then subcultured on PDA for three weeks. Bacterial and fungal subcultures were stored at 4°C until DNA isolation.

**Table 1:** Composition of the standard and selective media (based on one litre of medium) used to isolate soil bacteria. Each medium contained 100 mg / L of cycloheximide.

Medium	Agar (g)	TSB (g)	Micro-element ^d^ (ml)	Macro-element ^e^ (ml)	Diesel (ml)	Oil (ml)	Crude Oil (ml)	Acetone (ml)
1.5% TSA—1%OD ^a^	15	15			5	5		5
0.1% TSA—COC ^b^	15	1	1	10			0.2	0.1
0.1% TSA—COM ^c^	15	1	1	10			20	10
0.1% TSA—1%OD	15	1	1	10	5	5		5
0.1% TSA—5%OD	15	1	1	10	25	25		25
0.1% TSA—10%OD	15	1	1	10	50	50		50

^a^ Oil–Diesel engine oil (Rotella diesel engine oil, Shell, Montreal, QC).

^b^ Crude Oil was Coated onto the medium after solidification (from Gulf of Mexico, provided by Montreal pipeline).

^c^ Crude Oil was mixed with the growth medium (Montreal pipeline).

^d^ MgSO_4_ (739 mg / L), KNO_3_ (76 mg / L), KCl (65 mg / L), KH_2_PO_4_ (4.1 mg / L).

^e^ MnSO_4_*7H_2_O (6 mg / L), ZnSO_4_*7H_2_O (2.65 mg / L), H_3_BO_3_ (1.5 mg / L), CuSO_4_ (0.13 mg / L), Na_2_MoO_4_*2H_2_O (0.002 mg / L), KI (0.75 mg / L).

**Table 2:** Composition of the standard and selective media (based on one liter of medium) used to isolate soil fungi.

Medium	Agar (g)	PDB (g)	Micro-element (ml)	Macro-element (ml)	Diesel (ml)	Oil (ml)	Crude Oil (ml)	Acetone (ml)
1.2% PDA—1%OD	15	12			5	5		5
0.1% PDA—COC	15	1	1	10			0.2	0.1
0.1% PDA—COM	15	1	1	10			20	10
0.1% PDA—1%OD	15	1	1	10	5	5		5
0.1% PDA—5%OD	15	1	1	10	25	25		25
0.1% PDA—10%OD	15	1	1	10	50	50		50

Each medium contained 200 mg / L of streptomycin, and 100 mg / L of ampicillin.

### DNA isolation, amplification, and sequencing of microbial isolates

Bacterial strains were picked with a 1 μl sterile inoculation loop (Sarstedt, Montreal, Canada) and spiked directly into a PCR master mix (described below). Fungal strains were subcultured for one week in PDB before we harvested fresh mycelium for the isolation of genomic DNA (gDNA). All gDNA isolations were performed using a Freedom EVO100 extraction robot (Tecan Group, Mannedorf, Switzerland) with the NucleoMag 96 Plant kit (Macherey Nagel, Oesingen, Switzerland) according to the manufacturer’s instructions. Bacterial 16S rDNA and fungal ITS sequences were amplified using the 27F [43] / 1492R [44], and the ITS1-F [45] / ITS4 [46] primer sets, respectively. PCR mixtures were made up of 1× PCR buffer, 4% DMSO, 0.5 mg BSA, 0.5 mM MgCl_2_, 0.2 mM of each deoxynucleotide triphosphate, 0.5 μM of each primer, one unit of DreamTaq DNA polymerase (Fermentas, Canada) and one μl of gDNA (or picked colony) in a total volume of 50 μl. Thermal cycling conditions for bacteria were as follows: initial denaturation at 94°C for 5 min; 30 cycles at 94°C for 1 min, 55°C for 1 min, and 72°C for 1 min; and a final elongation at 72°C for 10 min. Thermal cycling conditions for fungi were as follows: initial denaturation at 94°C for 2 min; 35 cycles at 94°C for 30 s, 52°C for 30 s, and 72°C for 1 min; and a final elongation at 72°C for 5 min. PCR reactions were performed using an Eppendorf Mastercycler ProS (Eppendorf, Mississauga, ON), and products were visualized on GelRed-stained 1.5% agarose gels using the Gel-Doc system (Bio-Rad Laboratories, Mississauga, ON). DNA sequencing was performed on an Applied Biosystems 3730xl DNA analyzer (Applied Biosystems, Carlsbad, CA) at the McGill University and Genome Quebec Innovation Centre (Montreal, QC). Sanger sequencing data are deposited in GenBank under the accession numbers KP177318—KP177405 and KP177406—KP177454 for bacteria and fungi, respectively.

### Culture-independent (CI) sample processing

Total soil DNA was isolated from one gram of each composite soil sample using the MoBio PowerSoil DNA Isolation Kit following the manufacturer’s instructions. For each sample, triplicate PCR reactions of partial 16S rDNA and fungal ITS amplicons were performed using barcoded primers with the required 454 adapter sequences (S2A and S2B Table). Thermal cycling conditions for reactions using 16S rDNA barcoded primers were as follows: initial denaturation at 95°C for 5 min; 30 cycles at 95°C for 30 s, 55°C for 30 s, and 72°C for 30 s; and a final elongation at 72°C for 7 min. Thermal cycling conditions for reactions using ITS barcoded primers were as follows: initial denaturation at 94°C for 4 min; 30 cycles at 94°C for 30 s, 50°C for 60 s, and 72°C for 90 s; and a final elongation at 72°C for 10 min. PCR reactions were performed on an Eppendorf Mastercycler ProS (Eppendorf, Mississauga, ON). PCR triplicates were combined and quantified using the Quant-iT PicoGreen dsDNA assay kit (Invitrogen, Life Technologies). 16S rDNA and ITS pools from each of the five composite samples were then combined in equimolar ratios. High-throughput sequencing was performed on the 454 GS FLX+ platform using Lib-L chemistry (Roche, Branford, CT, USA). The 16S rDNA and ITS pools were each sequenced using ¼ of a plate. The 454-pyrosequencing data generated in this study have been deposited in the NCBI Sequence Read Archive and are available under the BioSample accession numbers SAMN03199986 and SAMN03199987.

### Bioinformatic analyses

Quality processing (read quality trimming, chimera check) of partial 16S rDNA and ITS1 sequences was performed in Mothur v.1.33.2 following the protocol used in Schloss et al. [47] and accessed online (http://www.mothur.org/wiki/454_SOP) in September 2014. Sequences obtained from Sanger sequencing were edited, cleaned, and assembled in Geneious Pro v.6.1.5 (Biomatters). For both 454 sequences and Sanger sequences, clustering analyses were performed in Geneious, and OTUs were defined at 97% similarity. For 454-pyrosequencing datasets, singletons were not considered, and doubletons with a nucleotide similarity <100% were excluded. OTU rarefaction curves, Venn diagrams, taxonomic identifications, relative abundance analyses, and richness and diversity indices were performed in Mothur. Taxonomic identifications for bacterial and fungal sequences were performed in Mothur using the SILVA [48] and UNITE [49] databases, respectively. Prior to performing community comparisons based on the 454-pyrosequencing datasets, each library was randomly subsampled to the minimum number of sequences observed per composite soil sample. Heatmaps and Venn diagrams were created using the R statistical language v.3.0.0 [50] with the packages gplots [51] and VennDiagram [52]. Krona charts were calculated using the KronaTools available from http://krona.sourceforge.net [53]. To partition the datasets between abundant and rare OTUs, the number of reads observed for each OTU was divided by the number of reads counted for the most abundant OTU. Rare OTUs were defined as those with a read proportion of < 5% (S1 Fig).

## Results

After quality trimming, OTU filtering, and standardizing the number of sequences per sample, 22,422 16S rDNA sequences and 10,098 ITS sequences were recovered from bacterial and fungal CI datasets, respectively (Table 3). OTU richness reached saturation at each level of contamination (S2A and S2B Fig), with the exception of plot 3 in the fungal CD dataset (coverage = 59.2%, Table 3). Using a similarity threshold of 97% to cluster sequences within the same OTU, a total of 2047 and 360 OTUs were recovered from bacterial and fungal CI datasets, respectively. For the culture-dependent (CD) datasets, 781 bacterial strains and 279 fungal strains were isolated and sequenced from the five plots, using the seven different culture media per microbial group. A total of 88 bacterial and 49 fungal OTUs were defined based on analysis of the 16S rDNA and ITS sequences, respectively.

**Table 3:** Bacterial and fungal OTU richness and diversity recovered in each library.

Method ^a^	Level of contamination ^b^	No. sequences	Coverage ^c^ (%)	Nb. OTUs	D ^d^	H ^e^	PD ^f^
**CI-Bacteria**	SC	9082	98.7	1342	658.4	6.8	57.9
	C	4519	94.1	1030	385.9	6.4	47.3
	HC	8821	98.6	887	135.1	5.9	47.2
**CI-Fungi**	SC	4017	99.5	235	35.1	4.3	32.1
	C	2033	98.5	144	16.5	3.6	19.4
	HC	4048	99.3	153	17.1	3.5	26.5
**CD-Bacteria**	SC	297	91.2	56	16.8	3.3	2.46
	C	172	93.6	30	9.0	2.7	1.36
	HC	312	98.7	36	13.0	3.0	1.47
**CD-Fungi**	SC	223	94.2	43	19.8	3.2	5.42
	C	27	59.2	16	15.2	2.5	2.35
	HC	29	89.6	8	4.3	1.6	1.12

^a^ CI: culture-independent (454-pyrosequencing); CD: culture-dependent (isolation).

^b^ SC: slightly contaminated; C: contaminated; HC: highly contaminated (see S1 Table).

^c^ Good's coverage.

^d^ Simpson’s inverse index of diversity.

^e^ Shannon index.

^f^ Faith’s phylogenetic diversity.

### Bacterial and fungal communities recovered by CI and CD methods

**Fig 1:**
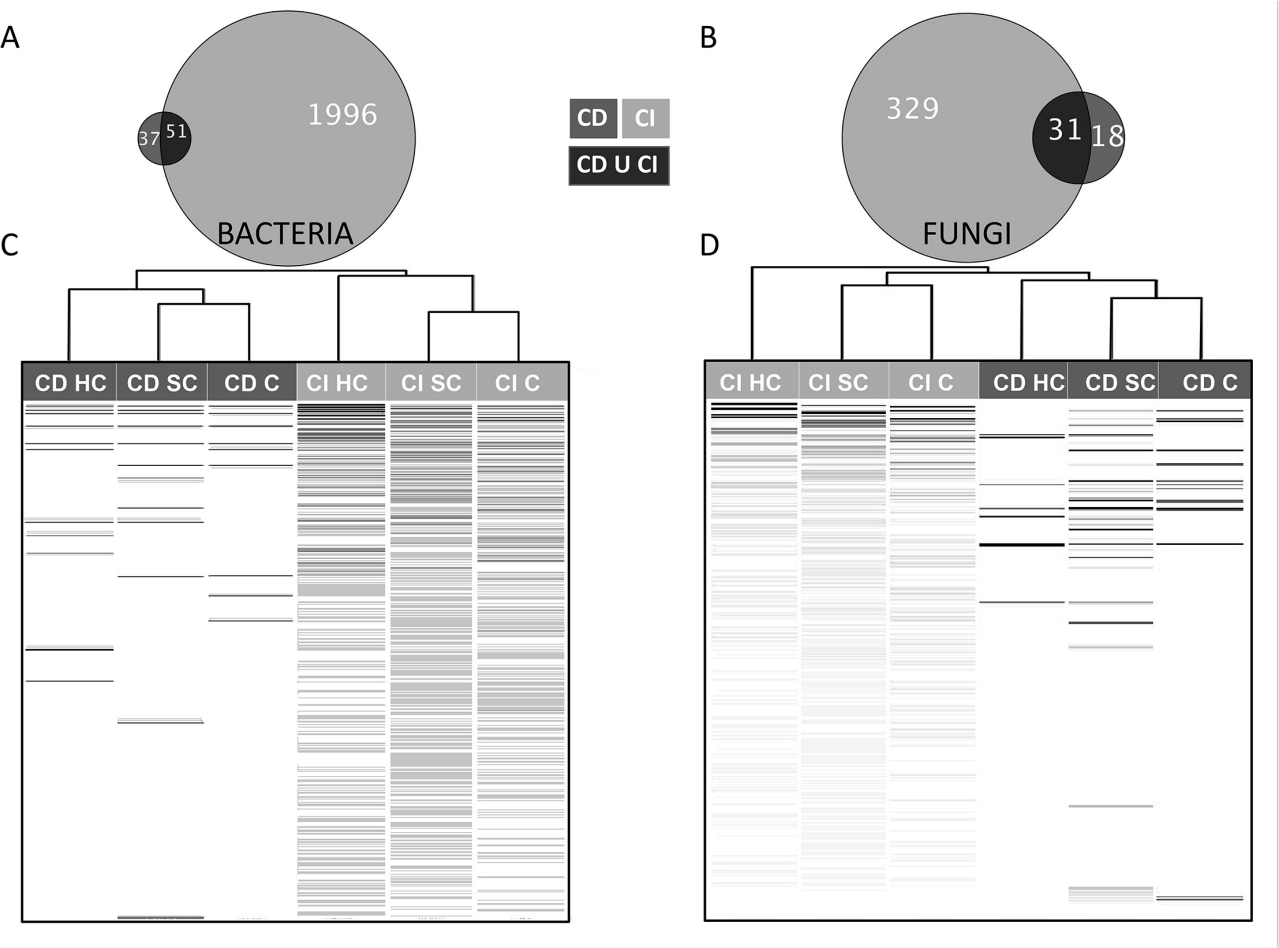
Proportional Venn diagram showing the distribution of bacterial (A) and fungal (B) OTUs between culture-independent (CI, light grey) and culture-dependent (CD, dark grey) methods. Heatmap distribution of the relative abundance of bacterial (C) and fungal (D) OTUs recorded with CI (light grey) and CD (dark grey) methods. OTUs are in rows and colour intensity indicates relative abundance, with black indicating the highest relative abundances observed for slightly contaminated (SC), contaminated (C) and highly contaminated (HC) composite soil sediments recovered with CI and CD methods. OTUs are presented by descending number of reads from top to bottom, based on proportion of total reads recorded in CI and CD datasets. The top dendrogram shows the hierarchical clustering of bacterial and fungal communities recovered for each PAH contamination level based on Bray-Curtis dissimilarity.

**Fig 2:**
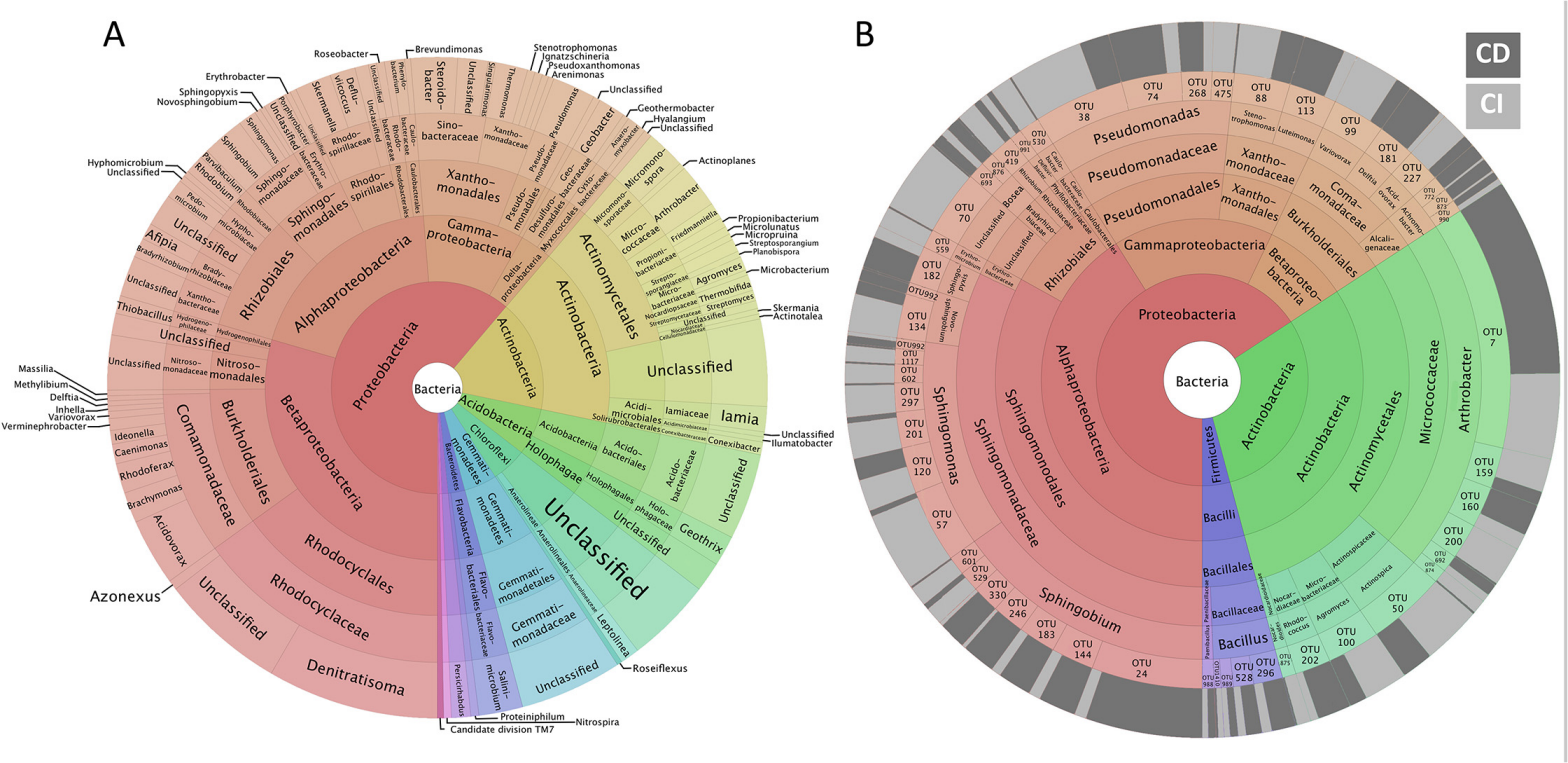
Krona charts showing the taxonomic identification and relative abundance of the most abundant bacterial OTUs recorded in CI datasets (A) and shared OTUs between CI and CD datasets (B). The proportion of sequences from the CI (light grey) or CD (dark grey) datasets for each shared OTU is shown in the outer ring.

The CI and CD methods provided two extremely divergent views of the microbial communities recorded in the five plots (Fig 1). The proportion of shared OTUs between CD and CI datasets was only 2.4% (51 OTUs) for bacteria and 8.2% (31 OTUs) for fungi (Fig 1A and 1B). This did, however, represent 58% and 63% of the bacterial and fungal strains isolated *in vitro*. Based on Bray-Curtis dissimilarity, bacterial and fungal communities from each contaminant level clustered first according to sampling method (due to the large difference in species richness between CI and CD datasets), and secondarily according to the level of hydrocarbon-contamination (Fig 1C and 1D), with the single exception of the CI HC fungal community which segregated independently from the other fungal assemblages. The heatmaps clearly show that the most abundant bacterial and fungal OTUs recovered via culturing do not correspond with those recovered by 454-pyrosequencing. *Proteobacteria* was the dominant bacterial phylum, representing 61% of the 16S rDNA reads from the 197 most abundant bacterial OTUs (Fig 2A). *Rhodocyclales* (15%), *Burkholderiales* (10%), *Actinomycetales* (10%), *Rhizobiales* (8%), *Xanthomonadales* (7%), and *Sphingomonadales* (5%) were the most frequently observed bacterial orders in the CI sequences (Fig 2A). Among the 51 bacterial OTUs shared between CI and CD methods (Fig 2B), OTU7 (*Arthrobacter* sp.) was the most frequently isolated, but represented the 61^st^ most abundant OTU out of 2047 in the CI dataset. Most of the shared OTUs belong to the genera *Sphingobium* and *Sphingomonas* (*Sphingomonodales*), *Pseudomonas* (*Pseudomonadales*) and *Arthrobacter* (*Actinomycetales*). *Sphingomonodales* and *Actinomycetales* were the most abundant orders identified within the shared OTUs, with relative abundances of 33% and 30% respectively, while accounting for 5% and 10% of reads in the CI dataset.

**Fig 3:**
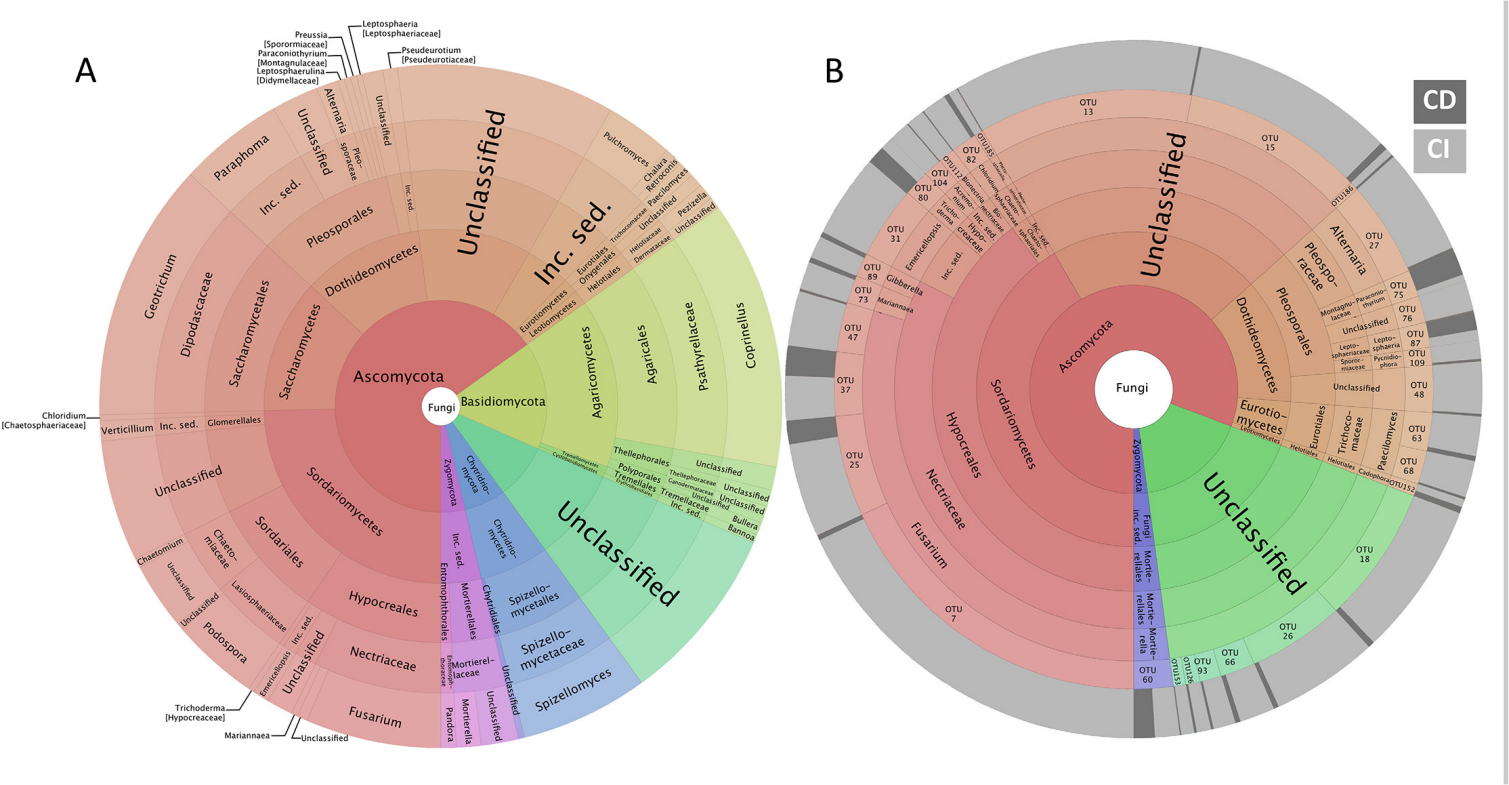
Krona charts showing the taxonomic identification and relative abundance of the most abundant fungal OTUs recorded in CI datasets (A) and shared OTUs between CI and CD datasets (B). The proportion of sequences from the CI (light grey) or CD (dark grey) datasets for each shared OTU is shown in the outer ring.

With respect to the fungal communities observed in the CI dataset, *Ascomycota* was the dominant phylum, representing 65% of all reads (Fig 3A), while no *Glomeromycota* sequence was recovered in the CI dataset. At the order level, *Agaricales* (13%), *Saccharomycetales* (12%), *Hypocreales* (10%), *Pleosporales* (9%), *Sordariales* (8%) and *Spizellomycetales* (6%) dominated the fungal community. Eighteen percent of the reads could not be identified. In contrast to the bacterial datasets, some fungal isolates ranked among the 20 most abundant OTUs in the CI dataset (Fig 3B). For example, OTU7, OTU13, and OTU15 were ranked seventh, thirteenth, and sixteenth in the CI dataset, respectively, based on read abundance. Only two isolated strains (OTU 75 and 87) were more frequently observed in the CD dataset than in the CI dataset (Fig 3B). A large proportion of the shared OTUs could not be identified (39% of all shared sequences), while *Hypocreales* (40%) and *Pleosporales* (10%) were the two prevalent fungal orders in the CD dataset. While some members of the *Basidiomycota* were recorded in the CI dataset, none were isolated.

Interestingly, some of the isolated bacterial and fungal strains could not be detected by 454-pyrosequencing (S3A and S3B Fig), despite the fact that the number of reads from the CI analysis was more than an order of magnitude higher than the number of sequences in CD datasets, and the rarefaction curves were saturated. *Sphingobium* represented 24% of the bacterial taxa that were found only in the CD dataset, followed by *Staphylococcus* (14%) and *Arthrobacter* (8%) (S3A Fig). For fungi, OTU81 and OTU108 (*Trichoderma*), and OTU97 and OTU118 (*Paraphoma*) represented 66% of the isolated strains that were not detected by 454-pyrosequencing (S3B Fig). The majority of non-shared bacterial and fungal OTUs were isolated on media containing oil-derived hydrocarbons (data not shown).

## Discussion

Culturing is essential to both the study and application of microbially-mediated bioremediation, yet the proportion and identity of the microbial community in hydrocarbon-contaminated soils that is culturable has remained unclear. Our results show that not only does culturing select a small proportion of the microorganisms that can be observed through CI methods, a fact that has been long known, but that many of the most abundant microorganisms detected using CI methods are not captured with plate-culturing techniques. This suggests that the microbes that are selected by culturing are not even representative of those that are highly competitive in the environment.

### Culture-independent versus culture-dependent methods

As shown by saturated rarefaction curves, the sampling effort for both CI and CD methods was adequate for capturing most of the abundant OTUs that could be identified with these methods in the five composite sediment samples. This allows us to draw robust comparisons between these datasets. Previous studies that have compared bacterial or fungal diversity using CI and CD approaches concluded that these methods were complementary [36,38,54–57]. In this study, the number of taxa that were unique to the CD dataset was limited, despite the use of seven different culture media for isolation. Taxa identified from isolation alone increased total species richness by only 2% for bacteria and 5% for fungi, meaning that 454-pyrosequencing captured 95% or more of the microbial diversity that could be obtained using these methods, and with far less effort. The OTUs that were only identified by culturing likely represent rare taxa that could not be observed at the sequencing depth used in this study. Extremely rare taxa may in fact represent a large proportion of the OTUs in natural microbial communities [58]. The presence of hydrocarbons in six out of the seven isolation media could explain the limited number of OTUs that we recovered through isolation, since hydrocarbon contaminants can negatively affect soil bacterial and fungal diversity [22,23,59]. Alternative culturing methods, such as the use of rhizosphere isolation medium [37], micro-cultivation techniques [40,60] and dilute nutrient media [25,61,62], have isolated a larger number of microorganisms, but the overlap with CI community analysis has been limited in all cases, and many OTUs that are highly abundant in CI datasets cannot be isolated. For instance Shade et al. [37] performed 454-pyrosequencing on total cultivated soil bacteria without colony picking, and reported that only 9.1% of bacterial OTUs were shared between CD and CI datasets, while each dataset contained 37,645 and 46,347 reads, representing a total of 4957 OTUs. As in our study, the authors demonstrated that many of the shared OTUs represented rare taxa in the CI dataset. Lagier et al. [36] investigated human gut bacteria by microbial culturomics (212 different culture conditions, yielding 32,500 colonies) and metagenomics, and in this case, only 5.1% of total OTUs were shared between sampling approaches. Furthermore, the authors identified 174 novel bacterial species in the CD dataset, demonstrating that metagenomics can be an inefficient method for detecting extremely rare bacteria.

Since many of the bacterial and fungal taxa that we isolated are not representative of the most abundant taxa *in situ*, their potential as effective *in situ* inoculants is questionable, even if some of these isolates demonstrated a high capacity for hydrocarbon degradation (unpublished data). Intermicrobial competition appears to play an important role in shaping microbial activity and abundance [63–65], and the persistence and activity of bioaugmented strains depends fundamentally on their ability to compete with indigenous microorganisms [66]. If strains isolated through culturing are only transient contributors to microbial community function, or exist permanently within the rare biosphere, they are unlikely to dominate when reinoculated, even if their abundance is maximized in the inoculum. This may be a major contributing factor to the poor efficiency of many prior attempts at bioaugmentation [66,67]. Spatial and temporal monitoring of microbial abundance in contaminated soils using CI methods appears to be a fundamental step towards determining the factors that govern the abundance of microorganisms *in situ*, as well as for selecting strains that can be successful across varied environments. However, if members of the rare biosphere are more efficient at hydrocarbon degradation than microbes that are naturally abundant, it may be desirable to develop vacant niches that allow these organisms to persist. This may be possible through the addition of plant hosts, for example [68].

### Comparing fungal and bacterial isolation from hydrocarbon-contaminated soils

A greater proportion of fungal OTUs identified via CI analysis were isolated from the hydrocarbon-contaminated sediments when compared with isolation of bacteria. This is likely due in part to the lower richness of fungi than bacteria in soil. While roughly 1000 fungal OTUs were identified in 4 grams of forest soil [69], between 6000–50,000 bacterial species can potentially be recovered from a single gram of soil, depending on where the “species” cutoff is drawn [70]. In addition, fungi and bacteria may be differentially sensitive to small changes in environmental conditions. Fungal propagules, such as hyphae and spores, are generally much larger than bacteria. As a result, certain soil microenvironments that are relevant to bacterial distribution may not affect fungi, as these organisms may develop niches at different spatial scales. Fungi have also been shown to be much less sensitive to pH variation than bacteria, which might allow cultivation of a wider range of fungi than bacteria using media at only one pH level [71]. On the other hand, fungi have been shown to be more sensitive to high hydrocarbon concentrations and were more strongly influenced by the presence of plants than bacteria in this study [23].

Still, only a fraction of known fungal species can be cultured (ranging from 5 to 17% according to [72–74]), while most species that belong to the phylum *Basidiomycota* cannot yet be isolated or grown under *in vitro* conditions. Taxa within the *Glomeromycota* cannot be cultivated at all without host plant roots. In our CI dataset, two taxa from the *Agaricomycetes* (*Basidiomycota*) ranked first and second in terms of read abundance, but were not captured by culturing efforts. These two OTUs have non-conspecific matches with ITS sequences of *Coprinellus*, a genus in the family *Psathyrellaceae*. Their absence in the CD dataset is somewhat surprising, since species of *Coprinellus* and *Coprinopsis* have been grown in Petri dishes [75]. Suhara et al. [75] reported near-complete degradation of 1 μmol of polychlorinated dibenzo-p-dioxin by *Coprinellus disseminatus* in two weeks. Minimal information is available with regards to the hydrocarbon-degrading capacity of members of this family, although *Coprinellus bisporus* was recorded, along with other fungi, from soil contaminated with phenanthrene and pyrene and treated with pea wheat straw [76]. The third most abundant OTU had a non-conspecific match with ITS sequences from the genus *Geotrichum*. *Geotrichum candidum* was previously found to increase benzo[a]pyrene degradation in mineral liquid medium [77]. Zheng & Obbard [78] isolated a strain identified as *Geotrichum* sp. in a PAH-contaminated soil, which was able to oxidize more than 50% of the phenanthrene present in liquid medium after seven days. The authors were able to isolate and cultivate this strain of *Geotrichum* sp. using generic media that were not hydrocarbon-coated, while the OTU observed at our site was not isolated, despite the use of both selective and non-selective media.

The three most abundant bacterial taxa in our CI dataset had non-conspecific matches in GenBank with environmental 16S rDNA sequences from heavy metal or oil-contaminated soils. The first two OTUs belong to the *Rhodocyclaceae* (*Denitratisoma* and unclassified taxon) while the third is a member of the *Comamonadaceae* (*Acidovorax*). PAH-degrading bacteria have been reported from both of these families [79–81]. In addition, members of the *Rhodocyclaceae* have been shown to degrade PAHs adsorbed in hydrophobic sorbents, highlighting the ability of these strains to degrade PAHs even when they are poorly bioavailable [80]. Thus, the most abundant bacterial and fungal taxa recorded by 454-pyrosequencing from this hydrocarbon-contaminated site belong to taxonomic groups that include members with the capacity to degrade hydrocarbons. Unfortunately, none of these taxa could be isolated using traditional culturing techniques, preventing comprehensive testing of the hydrocarbon-degrading potential of these strains.

### Conclusions

To date, this is the most comprehensive comparison of CI and CD methods in hydrocarbon-contaminated soils, and the first to examine fungal communities. Although microbial diversity can be reduced in contaminated environments, we did not recover a large proportion of the bacterial or fungal OTUs that were identified through 454-pyrosequencing. Surprisingly, many of the most abundant OTUs *in situ* were not cultured, despite the use of several types of hydrocarbon-containing media, suggesting that factors other than hydrocarbon tolerance and metabolism are critical to *in situ* community composition. Although obligate hydrocarbonoclastic microorganisms have been identified [82], they appear to be uncommon, and many microorganisms can adapt their existing metabolic pathways to the degradation of hydrocarbon compounds. Based on this, as well as our results, hydrocarbons may be a poor selective agent for comprehensively culturing representative microorganisms from hydrocarbon-contaminated soils, despite the widespread use of hydrocarbon-coated media for isolating strains from such environments. This suggests that novel culturing techniques are essential to the progression of bioremediation research. Although next-generation sequencing has caused CI analyses to wildly outpace our ability to culture microorganisms, novel ‘omics’ tools will be invaluable in developing future generations of culturing methods, as some of the environmental and ecological factors that shape microbial abundance can be identified and exploited.

## Supporting Information

S1 Fig Rank abundance distribution of bacterial and fungal OTUs recorded in CI datasets. Singletons and doubletons with a pairwise similarity not equal to 100% are omitted. The dashed lines represent the partition between abundant and rare OTUs. A total of 197 bacterial OTUs were defined as abundant, representing 39.4% of the 16S rDNA reads, while 87 fungal OTUs were recognised as abundant representing 81.4% of the ITS reads.
(DOCX)

S2 Fig OTU rarefaction curves based on 454-pyrosequencing of bacteria (A) and fungi (B) and on Sanger sequencing of the isolated bacterial strains (C) and fungal strains (D) in the five plots sampled, combined according to hydrocarbon contamination level. The green, orange, and red curves show data observed in soil samples from the slightly contaminated, moderately contaminated, and highly contaminated plots, respectively.
(DOCX)

S3 Fig Krona charts showing the taxonomic identification and relative abundance of bacterial (A) and fungal (B) taxa recovered by CD methods. (DOCX)

S1 Table Polycyclic aromatic hydrocarbons (PAHs) and C10-C50 hydrocarbons recorded in the five plots sampled. (DOCX)

S2 Table A. Fusion primers used to amplify bacterial taxa from the five plots sampled using 454-pyrosequencing. Adapters 1 and 2 were CCATCTCATCCCTGCGTGTCTCCGAC and CCTATCCCCTGTGTGCCTTGGCAGTC, respectively, followed by the key sequence TCAG. **B. Fusion primers used to amplify fungal communities from the five plots sampled using 454-pyrosequencing.** Adapters 1 and 2 were CCATCTCATCCCTGCGTGTCTCCGAC and CCTATCCCCTGTGTGCCTTGGCAGTC, respectively, followed by the key sequence TCAG.
(DOCX)
