# Supplementary material for: Culture-Dependent and -Independent Methods Capture Different Microbial Community Fractions in Hydrocarbon-Contaminated Soils
Source: PLoS One. 2015 Jun 8;10(6):e0128272. doi: 10.1371/journal.pone.0128272 (PMC4460130; doi:10.1371/journal.pone.0128272)
Supplement: S1 Fig — Singletons and doubletons with a pairwise similarity not equal to 100% are omitted. The dashed lines represent the partition between abundant and rare OTUs. A total of 197 bacterial OTUs were defined as abundant, representing 39.4% of the 16S rDNA reads, while 87 fungal OTUs were recognised as abundant representing 81.4% of the ITS reads. (DOCX) [file pone.0128272.s001.docx]

**Supporting Information**

# Figure S1.
