# Supplementary material for: Culture-Dependent and -Independent Methods Capture Different Microbial Community Fractions in Hydrocarbon-Contaminated Soils
Source: PLoS One. 2015 Jun 8;10(6):e0128272. doi: 10.1371/journal.pone.0128272 (PMC4460130; doi:10.1371/journal.pone.0128272)
Supplement: S2 Fig — The green, orange, and red curves show data observed in soil samples from the slightly contaminated, moderately contaminated, and highly contaminated plots, respectively. (DOCX) [file pone.0128272.s002.docx]

**Supporting Information**

# Figure S2.
