# Supplementary figures and images for: Culture-Dependent and -Independent Methods Capture Different Microbial Community Fractions in Hydrocarbon-Contaminated Soils
Source: PLoS One. 2015 Jun 8;10(6):e0128272. doi: 10.1371/journal.pone.0128272 (PMC4460130; doi:10.1371/journal.pone.0128272)

**Supporting Information**

**
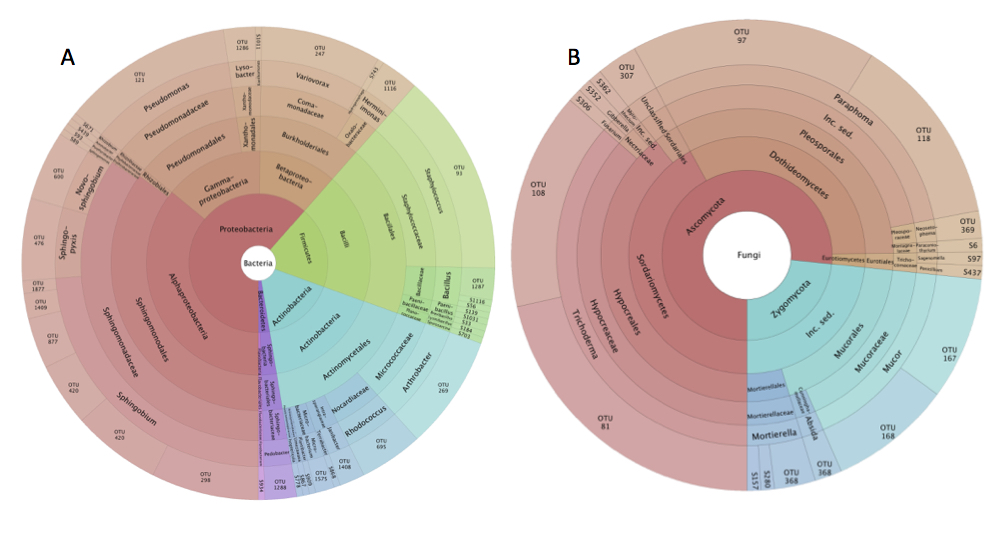
** Figure S3.

Supplement: S3 Fig — (DOCX) [file pone.0128272.s003.docx]
