# Supplementary material for: Culture-Dependent and -Independent Methods Capture Different Microbial Community Fractions in Hydrocarbon-Contaminated Soils
Source: PLoS One. 2015 Jun 8;10(6):e0128272. doi: 10.1371/journal.pone.0128272 (PMC4460130; doi:10.1371/journal.pone.0128272)
Supplement: S1 Table — (DOCX) [file pone.0128272.s004.docx]

**Supporting Information**

Table S1. Polycyclic aromatic hydrocarbons (PAHs) and C10-C50 hydrocarbons recorded in the five plots sampled.

| PAHs and C10-C15 hydrocarbons | Plot 1 | Plot 2 | Plot 3 | Plot 4 | Plot 5 |
| --- | --- | --- | --- | --- | --- |
| Acenaphthene (mg/kg) | 0 | 0 | 16.9 | 110 | 110 |
| Acenaphthylene (mg/kg) | 0.1 | 0.1 | 1.3 | 6.5 | 5.6 |
| Anthracene (mg/kg) | 0.1 | 0.4 | 9.9 | 53.6 | 45.5 |
| Benzo[a]anthracene (mg/kg) | 0 | 0 | 0.8 | 3.3 | 2.4 |
| Benzo[a]pyrene (mg/kg) | 0 | 0 | 0.2 | 0.7 | 0.4 |
| Benzo[b,j,k]fluoranthene (mg/kg) | 0 | 0 | 0.2 | 0.9 | 0.6 |
| Benzo[c]phenanthrene (mg/kg) | 0 | 0 | 0.3 | 1.4 | 1 |
| Benzo(g,h,i)perylene (mg/kg) | 0 | 0 | 0 | 0 | 0 |
| Chrysene (mg/kg) | 0 | 0 | 1 | 5.2 | 3.2 |
| Dibenzo[a,h]anthracene (mg/kg) | 0 | 0 | 0 | 0 | 0 |
| Dibenzo[a,i]pyrene (mg/kg) | 0 | 0 | 0 | 0 | 0 |
| Dibenzo[a,h]pyrene (mg/kg) | 0 | 0 | 0 | 0 | 0 |
| Dibenzo[a,l]pyrene (mg/kg) | 0 | 0 | 0 | 0 | 0 |
| 7,12-Dimethylbenz[a]anthracene (mg/kg) | 0 | 0 | 0 | 0 | 0 |
| Fluoranthene (mg/kg) | 0 | 0 | 3.5 | 18.3 | 14 |
| Fluorene (mg/kg) | 0 | 0 | 11.3 | 85.5 | 90.2 |
| Indeno[1,2,3-cd]pyrene (mg/kg) | 0 | 0 | 0 | 0 | 0 |
| 3-Methylcholanthrene (mg/kg) | 0 | 0 | 0 | 0 | 0 |
| Naphthalene (mg/kg) | 0 | 0 | 6.3 | 59.6 | 93 |
| Phenanthrene (mg/kg) | 0.1 | 0.2 | 60.6 | 318 | 314 |
| Pyrene (mg/kg) | 0 | 0.1 | 5.2 | 27.4 | 20.7 |
| 1-Methylnaphthalene (mg/kg) | 0 | 0 | 14.5 | 81.9 | 85.9 |
| 2-Methylnaphthalene (mg/kg) | 0 | 0 | 14.6 | 71.2 | 91.3 |
| 1-3-Dimethylnaphthalene (mg/kg) | 0 | 0 | 23.4 | 92.2 | 69,0 |
| 2,3,5-trimethylnaphthalene (mg/kg) | 0 | 0 | 6.6 | 28.2 | 21.6 |
| Acenaphthene-D10 (%) | 104 | 117 | 134 | 94 | 107 |
| Fluoranthene-D10 (%) | 102 | 121 | 104 | 126 | 110 |
| Perylene-D12 (%) | 112 | 115 | 93 | 103 | 93 |
| Hydrocarbons C10 - C50 (mg/kg) | <100 | <100 | 2870 | 6400 | 5900 |
