# Supplementary material for: Culture-Dependent and -Independent Methods Capture Different Microbial Community Fractions in Hydrocarbon-Contaminated Soils
Source: PLoS One. 2015 Jun 8;10(6):e0128272. doi: 10.1371/journal.pone.0128272 (PMC4460130; doi:10.1371/journal.pone.0128272)
Supplement: S2 Table — Adapters 1 and 2 were CCATCTCATCCCTGCGTGTCTCCGAC and CCTATCCCCTGTGTGCCTTGGCAGTC, respectively, followed by the key sequence TCAG. B. Fusion primers used to amplify fungal communities from the five plots sampled using 454-pyrosequencing. Adapters 1 and 2 were CCATCTCATCCCTGCGTGTCTCCGAC and CCTATCCCCTGTGTGCCTTGGCAGTC, respectively, followed by the key sequence TCAG. (DOCX) [file pone.0128272.s005.docx]

**Supporting Information**

# Table S2A. Fusion primers used to amplify bacterial communities from the five plots sampled for 454-pyrosequencing. Adapters 1 and 2 were CCATCTCATCCCTGCGTGTCTCCGAC and CCTATCCCCTGTGTGCCTTGGCAGTC, respectively, followed by the key sequence TCAG.

| Composite soil sample | MID sequence | 27F  (Lane 1991) | 1492R (I)  (Turner *et al.* 1999) |
| --- | --- | --- | --- |
| Plot 1 | ACGAGTGCGT | AGAGTTTGATCMTGGCTCAG | GGTTACCTTGTTACGACTT |
| Plot 2 | ACGCTCGACA | AGAGTTTGATCMTGGCTCAG | GGTTACCTTGTTACGACTT |
| Plot 3 | AGACGCACTC | AGAGTTTGATCMTGGCTCAG | GGTTACCTTGTTACGACTT |
| Plot 4 | AGCACTGTAG | AGAGTTTGATCMTGGCTCAG | GGTTACCTTGTTACGACTT |
| Plot 5 | ATCAGACACG | AGAGTTTGATCMTGGCTCAG | GGTTACCTTGTTACGACTT |

**Table S2B. Fusion primers used to amplify fungal communities from the five plots sampled for 454-pyrosequencing.** Adapters 1 and 2 were CCATCTCATCCCTGCGTGTCTCCGAC and CCTATCCCCTGTGTGCCTTGGCAGTC, respectively, followed by the key sequence TCAG.

| Composite soil sample | MID sequence | ITS1  (White *et al.* 1990) | ITS4  (White *et al.* 1990) |
| --- | --- | --- | --- |
| Plot 1 | CTCGCGTGTC | TCCGTAGGTGAACCTGCGG | TCCTCCGCTTATTGATATGC |
| Plot 2 | TCTCTATGCG | TCCGTAGGTGAACCTGCGG | TCCTCCGCTTATTGATATGC |
| Plot 3 | TACTGAGCTA | TCCGTAGGTGAACCTGCGG | TCCTCCGCTTATTGATATGC |
| Plot 4 | CATAGTAGTG | TCCGTAGGTGAACCTGCGG | TCCTCCGCTTATTGATATGC |
| Plot 5 | CGAGAGATAC | TCCGTAGGTGAACCTGCGG | TCCTCCGCTTATTGATATGC |
